# 2-Chloro­phenyl 4-methyl­benzoate

**DOI:** 10.1107/S1600536808022290

**Published:** 2008-07-19

**Authors:** B. Thimme Gowda, Sabine Foro, K. S. Babitha, Hartmut Fuess

**Affiliations:** a Department of Chemistry, Mangalore University, Mangalagangotri 574 199, Mangalore, India; b Institute of Materials Science, Darmstadt University of Technology, Petersenstrasse 23, D-64287 Darmstadt, Germany

## Abstract

The conformation of the C=O bond in the title compound, C_14_H_11_ClO_2_, is *anti* to the Cl atom, similar to what was observed in 2-methyl­phenyl 4-methyl­benzoate. The dihedral angle between the two aromatic rings is 59.36 (7)°.

## Related literature

For related literature, see: Gowda *et al.* (2008*a* ▶,*b* ▶,*c* ▶); Nayak & Gowda (2008 ▶).
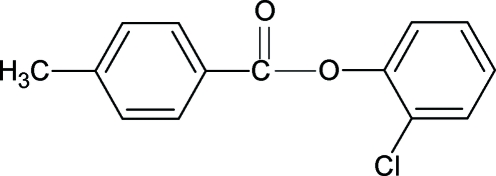


## Experimental

### 

#### Crystal data

C_14_H_11_ClO_2_
*M* *_r_* = 246.68
Monoclinic, 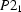

*a* = 4.0538 (8) Å
*b* = 13.661 (3) Å
*c* = 10.975 (2) Å
β = 91.70 (2)°
*V* = 607.5 (2) Å^3^
*Z* = 2
Mo *K*α radiation
μ = 0.30 mm^−1^
*T* = 299 (2) K
0.48 × 0.24 × 0.16 mm

#### Data collection

Oxford Diffraction Xcalibur diffractometer with a Sapphire CCD detector
Absorption correction: multi-scan (*CrysAlis RED*; Oxford Diffraction, 2007 ▶) *T*_min_ = 0.869, *T*_max_ = 0.954
4160 measured reflections
2238 independent reflections
1632 reflections with *I* > 2σ(*I*)
*R*_int_ = 0.012

#### Refinement

*R*[*F* ^2^ > 2σ(*F* ^2^)] = 0.035
*wR*(*F* ^2^) = 0.116
*S* = 1.15
2238 reflections
155 parameters
1 restraint
H-atom parameters constrained
Δρ_max_ = 0.36 e Å^−3^
Δρ_min_ = −0.34 e Å^−3^
Absolute structure: Flack (1983 ▶), 957 Friedel pairs
Flack parameter: −0.09 (9)

### 

Data collection: *CrysAlis CCD* (Oxford Diffraction, 2004 ▶); cell refinement: *CrysAlis RED* (Oxford Diffraction, 2007 ▶); data reduction: *CrysAlis RED*; program(s) used to solve structure: *SHELXS97* (Sheldrick, 2008 ▶); program(s) used to refine structure: *SHELXL97* (Sheldrick, 2008 ▶); molecular graphics: *PLATON* (Spek, 2003 ▶); software used to prepare material for publication: *SHELXL97*.

## Supplementary Material

Crystal structure: contains datablocks I, global. DOI: 10.1107/S1600536808022290/bt2750sup1.cif

Structure factors: contains datablocks I. DOI: 10.1107/S1600536808022290/bt2750Isup2.hkl

Additional supplementary materials:  crystallographic information; 3D view; checkCIF report

## Figures and Tables

**Fig. 1.**
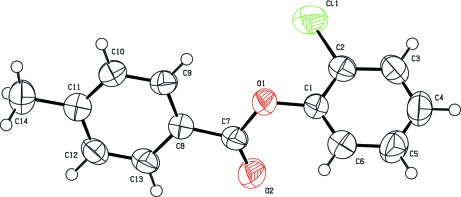
Molecular structure of the title compound, showing the atom labeling scheme. The displacement ellipsoids are drawn at the 50% probability level. The H atoms are represented as small spheres of arbitrary radii.

**Fig. 2.**
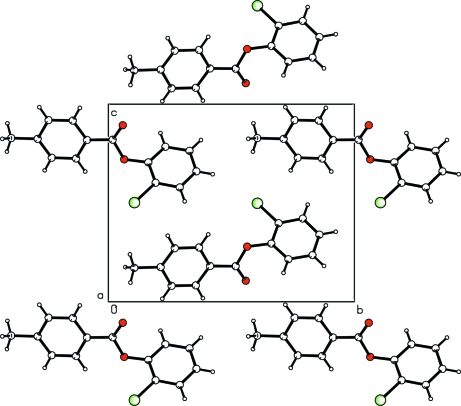
Packing diagram of the title compound.

## supplementary crystallographic information

### Comment

As part of a study of the substituent effects on the crystal structures of aryl benzoates (Gowda *et al.*, 2008*a*, 2008*b*, 2008*c*), in the present work, the structure of 2-chlorophenyl 4-methylbenzoate (2CP4MBA) has been determined. The conformation of the C=O bond in 2CP4MBA is *anti* to the *ortho*-chloro group in the phenolic benzene ring (Fig. 1), similar to what is observed in 2-methylphenyl 4-methylbenzoate (2MP4MBA) (Gowda *et al.*, 2008*c*). The dihedral angle between the benzene and benzoyl rings in 2CP4MBA is 59.36 (7)°, compared with the values of 71.75 (7)° in 3CP4MBA (Gowda *et al.*, 2008*a*), 63.89 (8)° in 4CP4MBA (Gowda *et al.*, 2008*b*) and 73.04 (8)° in 2MP4MBA (Gowda *et al.*, 2008*c*). Further, the bond parameters in 2CP4MBA are similar to those in 2MP4MBA and other aryl benzoates (Gowda *et al.*, 2008*a, b, c*). The packing diagram  is shown in Fig. 2.

### Experimental

The title compound was prepared according to a method of Nayak & Gowda (2008). The purity of the compound was checked by determining its melting point. It was characterized by recording its infrared and NMR spectra (Nayak & Gowda, 2008). Single crystals used in X-ray diffraction studies were obtained by slow evaporation of ethanolic solution of the title compound.

### Refinement

The H atoms were positioned with idealized geometry and refined using a riding model with C_aromatic_—H = 0.93Å or C_methyl_—H = 0.96 Å and with isotropic displacement parameters set to 1.2 *U*_eq_(C) or 1.5*U*_eq_(C_methyl_). The methyl group was allowed to rotate but not to tip.

### Crystal data

**Table:**

C_14_H_11_ClO_2_	*F*_000_ = 256
*M**_r_* = 246.68	*D*_x_ = 1.349 Mg m^−^^3^
Monoclinic, *P*2_1_	Mo *K*α radiation λ = 0.71073 Å
Hall symbol: P 2yb	Cell parameters from 1136 reflections
*a* = 4.0538 (8) Å	θ = 2.4–27.8º
*b* = 13.661 (3) Å	µ = 0.30 mm^−^^1^
*c* = 10.975 (2) Å	*T* = 299 (2) K
β = 91.70 (2)º	Long needle, colourless
*V* = 607.5 (2) Å^3^	0.48 × 0.24 × 0.16 mm
*Z* = 2	

### Data collection

**Table:**

Oxford Diffraction Xcalibur diffractometer with a Sapphire CCD detector	2238 independent reflections
Radiation source: fine-focus sealed tube	1632 reflections with *I* > 2σ(*I*)
Monochromator: graphite	*R*_int_ = 0.012
*T* = 299(2) K	θ_max_ = 26.4º
Rotation method data acquisition using ω and φ scans	θ_min_ = 2.4º
Absorption correction: multi-scan(CrysAlis RED; Oxford Diffraction, 2007)	*h* = −5→5
*T*_min_ = 0.869, *T*_max_ = 0.954	*k* = −17→15
4160 measured reflections	*l* = −13→7

### Refinement

**Table:**

Refinement on *F*^2^	Hydrogen site location: inferred from neighbouring sites
Least-squares matrix: full	H-atom parameters constrained
*R*[*F*^2^ > 2σ(*F*^2^)] = 0.035	*w* = 1/[σ^2^(*F*_o_^2^) + (0.0644*P*)^2^ + 0.0085*P*] where *P* = (*F*_o_^2^ + 2*F*_c_^2^)/3
*wR*(*F*^2^) = 0.116	(Δ/σ)_max_ = 0.018
*S* = 1.15	Δρ_max_ = 0.36 e Å^−^^3^
2238 reflections	Δρ_min_ = −0.34 e Å^−^^3^
155 parameters	Extinction correction: none
1 restraint	Absolute structure: Flack (1983), 957 Friedel pairs
Primary atom site location: structure-invariant direct methods	Flack parameter: −0.09 (9)
Secondary atom site location: difference Fourier map	

### Special details

**Table:**

Geometry. All e.s.d.'s (except the e.s.d. in the dihedral angle between two l.s. planes) are estimated using the full covariance matrix. The cell e.s.d.'s are taken into account individually in the estimation of e.s.d.'s in distances, angles and torsion angles; correlations between e.s.d.'s in cell parameters are only used when they are defined by crystal symmetry. An approximate (isotropic) treatment of cell e.s.d.'s is used for estimating e.s.d.'s involving l.s. planes.
Refinement. Refinement of *F*^2^ against ALL reflections. The weighted *R*-factor *wR* and goodness of fit *S* are based on *F*^2^, conventional *R*-factors *R* are based on *F*, with *F* set to zero for negative *F*^2^. The threshold expression of *F*^2^ > σ(*F*^2^) is used only for calculating *R*-factors(gt) *etc*. and is not relevant to the choice of reflections for refinement. *R*-factors based on *F*^2^ are statistically about twice as large as those based on *F*, and *R*- factors based on ALL data will be even larger.

### Fractional atomic coordinates and isotropic or equivalent isotropic displacement parameters (Å^2^)

**Table:**

	*x*	*y*	*z*	*U*_iso_*/*U*_eq_	
C1	0.7402 (8)	0.6625 (2)	0.2945 (2)	0.0525 (7)	
C2	0.5813 (7)	0.6919 (2)	0.3974 (2)	0.0570 (8)	
C3	0.5373 (9)	0.7904 (3)	0.4209 (3)	0.0699 (10)	
H3	0.4350	0.8104	0.4914	0.084*	
C4	0.6454 (10)	0.8581 (3)	0.3396 (3)	0.0801 (11)	
H4	0.6107	0.9243	0.3544	0.096*	
C5	0.8047 (9)	0.8300 (3)	0.2364 (4)	0.0751 (10)	
H5	0.8796	0.8765	0.1819	0.090*	
C6	0.8512 (9)	0.7319 (3)	0.2154 (3)	0.0677 (8)	
H6	0.9600	0.7122	0.1461	0.081*	
C7	0.6657 (7)	0.5173 (2)	0.1796 (2)	0.0513 (7)	
C8	0.7526 (7)	0.4132 (2)	0.1815 (2)	0.0460 (6)	
C9	0.9317 (7)	0.3687 (2)	0.2745 (2)	0.0527 (7)	
H9	1.0084	0.4057	0.3406	0.063*	
C10	0.9986 (7)	0.2701 (2)	0.2709 (3)	0.0579 (7)	
H10	1.1216	0.2417	0.3345	0.069*	
C11	0.8878 (7)	0.2126 (2)	0.1756 (3)	0.0548 (7)	
C12	0.7055 (7)	0.2569 (2)	0.0806 (2)	0.0562 (8)	
H12	0.6276	0.2192	0.0153	0.067*	
C13	0.6405 (7)	0.3554 (2)	0.0827 (2)	0.0552 (7)	
H13	0.5216	0.3841	0.0184	0.066*	
C14	0.9603 (8)	0.1054 (3)	0.1717 (3)	0.0726 (8)	
H14A	1.1020	0.0880	0.2399	0.087*	
H14B	1.0677	0.0900	0.0973	0.087*	
H14C	0.7577	0.0693	0.1754	0.087*	
O1	0.7986 (5)	0.56428 (14)	0.27927 (18)	0.0615 (6)	
O2	0.4938 (7)	0.55841 (16)	0.1064 (2)	0.0776 (7)	
Cl1	0.4406 (2)	0.60598 (9)	0.49831 (7)	0.0834 (3)	

### Atomic displacement parameters (Å^2^)

**Table:**

	*U*^11^	*U*^22^	*U*^33^	*U*^12^	*U*^13^	*U*^23^
C1	0.0659 (17)	0.0460 (19)	0.0452 (14)	0.0061 (14)	−0.0050 (12)	−0.0011 (12)
C2	0.0682 (19)	0.055 (2)	0.0468 (15)	−0.0031 (15)	−0.0084 (13)	−0.0042 (13)
C3	0.088 (2)	0.067 (3)	0.0541 (17)	0.0134 (19)	−0.0046 (16)	−0.0151 (16)
C4	0.103 (3)	0.047 (2)	0.089 (2)	0.008 (2)	−0.022 (2)	−0.008 (2)
C5	0.092 (3)	0.050 (2)	0.082 (2)	−0.0120 (19)	−0.0133 (19)	0.0066 (17)
C6	0.083 (2)	0.061 (2)	0.0597 (16)	−0.0017 (17)	−0.0004 (15)	−0.0015 (15)
C7	0.0616 (17)	0.0522 (19)	0.0397 (12)	−0.0047 (14)	−0.0037 (12)	−0.0034 (13)
C8	0.0517 (15)	0.0460 (18)	0.0405 (12)	−0.0052 (12)	0.0039 (11)	−0.0023 (11)
C9	0.0592 (18)	0.054 (2)	0.0442 (12)	−0.0002 (14)	−0.0031 (12)	0.0047 (12)
C10	0.0649 (18)	0.055 (2)	0.0529 (15)	0.0061 (15)	−0.0060 (13)	0.0039 (13)
C11	0.0583 (15)	0.046 (2)	0.0602 (16)	−0.0030 (14)	0.0119 (13)	−0.0024 (12)
C12	0.0686 (18)	0.057 (2)	0.0431 (14)	−0.0091 (15)	0.0048 (13)	−0.0107 (13)
C13	0.0638 (18)	0.0587 (19)	0.0427 (12)	−0.0029 (16)	−0.0052 (12)	−0.0060 (14)
C14	0.080 (2)	0.054 (2)	0.084 (2)	0.000 (2)	0.0041 (16)	−0.0060 (19)
O1	0.0858 (15)	0.0461 (12)	0.0514 (11)	0.0094 (10)	−0.0167 (10)	−0.0022 (8)
O2	0.1052 (17)	0.0570 (14)	0.0686 (13)	0.0119 (13)	−0.0286 (13)	−0.0011 (11)
Cl1	0.1042 (7)	0.0856 (6)	0.0608 (4)	−0.0113 (6)	0.0082 (4)	0.0093 (4)

### Geometric parameters (Å, °)

**Table:**

C1—C6	1.370 (5)	C8—C9	1.376 (4)
C1—O1	1.374 (3)	C8—C13	1.406 (4)
C1—C2	1.376 (4)	C9—C10	1.375 (4)
C2—C3	1.382 (4)	C9—H9	0.9300
C2—Cl1	1.723 (3)	C10—C11	1.373 (4)
C3—C4	1.366 (5)	C10—H10	0.9300
C3—H3	0.9300	C11—C12	1.398 (4)
C4—C5	1.375 (6)	C11—C14	1.495 (5)
C4—H4	0.9300	C12—C13	1.372 (4)
C5—C6	1.373 (5)	C12—H12	0.9300
C5—H5	0.9300	C13—H13	0.9300
C6—H6	0.9300	C14—H14A	0.9600
C7—O2	1.188 (3)	C14—H14B	0.9600
C7—O1	1.365 (3)	C14—H14C	0.9600
C7—C8	1.465 (4)		
			
C6—C1—O1	122.5 (3)	C13—C8—C7	117.5 (2)
C6—C1—C2	119.3 (3)	C10—C9—C8	120.8 (3)
O1—C1—C2	118.1 (3)	C10—C9—H9	119.6
C1—C2—C3	120.2 (3)	C8—C9—H9	119.6
C1—C2—Cl1	120.1 (2)	C11—C10—C9	121.5 (3)
C3—C2—Cl1	119.7 (2)	C11—C10—H10	119.3
C4—C3—C2	119.4 (3)	C9—C10—H10	119.3
C4—C3—H3	120.3	C10—C11—C12	118.3 (3)
C2—C3—H3	120.3	C10—C11—C14	121.5 (3)
C3—C4—C5	121.1 (4)	C12—C11—C14	120.2 (3)
C3—C4—H4	119.5	C13—C12—C11	120.6 (2)
C5—C4—H4	119.5	C13—C12—H12	119.7
C6—C5—C4	118.8 (4)	C11—C12—H12	119.7
C6—C5—H5	120.6	C12—C13—C8	120.4 (2)
C4—C5—H5	120.6	C12—C13—H13	119.8
C1—C6—C5	121.3 (3)	C8—C13—H13	119.8
C1—C6—H6	119.4	C11—C14—H14A	109.5
C5—C6—H6	119.4	C11—C14—H14B	109.5
O2—C7—O1	121.9 (3)	H14A—C14—H14B	109.5
O2—C7—C8	127.2 (2)	C11—C14—H14C	109.5
O1—C7—C8	110.8 (2)	H14A—C14—H14C	109.5
C9—C8—C13	118.3 (3)	H14B—C14—H14C	109.5
C9—C8—C7	124.1 (2)	C7—O1—C1	119.4 (2)
			
C6—C1—C2—C3	−0.6 (4)	C13—C8—C9—C10	−0.2 (4)
O1—C1—C2—C3	175.5 (3)	C7—C8—C9—C10	178.8 (3)
C6—C1—C2—Cl1	−179.8 (2)	C8—C9—C10—C11	−0.5 (4)
O1—C1—C2—Cl1	−3.7 (4)	C9—C10—C11—C12	0.5 (4)
C1—C2—C3—C4	1.7 (5)	C9—C10—C11—C14	−180.0 (3)
Cl1—C2—C3—C4	−179.1 (3)	C10—C11—C12—C13	0.2 (4)
C2—C3—C4—C5	−1.7 (5)	C14—C11—C12—C13	−179.3 (3)
C3—C4—C5—C6	0.6 (5)	C11—C12—C13—C8	−0.9 (4)
O1—C1—C6—C5	−176.4 (3)	C9—C8—C13—C12	0.9 (4)
C2—C1—C6—C5	−0.4 (5)	C7—C8—C13—C12	−178.2 (3)
C4—C5—C6—C1	0.4 (5)	O2—C7—O1—C1	−1.7 (4)
O2—C7—C8—C9	−174.5 (3)	C8—C7—O1—C1	−179.1 (3)
O1—C7—C8—C9	2.7 (4)	C6—C1—O1—C7	−64.4 (4)
O2—C7—C8—C13	4.5 (5)	C2—C1—O1—C7	119.6 (3)
O1—C7—C8—C13	−178.3 (2)		
